# Fecal Microbiota Transplantation Donor Screening: Is *Dientamoeba fragilis* a Valid Criterion for Donor Exclusion? A Longitudinal Study of a Swiss Cohort

**DOI:** 10.3390/microorganisms14010217

**Published:** 2026-01-17

**Authors:** Keyvan Moser, Aurélie Ballif, Trestan Pillonel, Maura Concu, Elena Montenegro-Borbolla, Beatrice Nickel, Camille Stampfli, Marie-Therese Ruf, Maxime Audry, Nathalie Kapel, Susanna Gerber, Damien Jacot, Claire Bertelli, Tatiana Galpérine

**Affiliations:** 1 Service of Infectious Diseases, Lausanne University Hospital, University of Lausanne, Rue du Bugnon 46, 1011 Lausanne, Switzerland; 2 Department of Policlinics, Centre for Primary Care and Public Health (Unisanté), 1010 Lausanne, Switzerland; 3 Institute of Microbiology, Lausanne University Hospital, University of Lausanne, 1011 Lausanne, Switzerland; 4 Swiss Tropical and Public Health Institute, 4123 Allschwil, Switzerland; 5 Diagnostic Center, Department of Medicine, University of Basel, 4002 Basel, Switzerland; 6 Service of Pharmacy, Lausanne University Hospital, University of Lausanne, 1011 Lausanne, Switzerland; 7 French Group for Fecal Microbiota Transplantation (GFTF), 75012 Paris, France; 8 Laboratoire de Coprologie Fonctionnelle, Hôpitaux Universitaires Pitié-Salpêtrière, Assistance Publique—Hôpitaux de Paris (APHP), 75651 Paris, France; 9 INSERM S1139, Université Paris Cité, 75006 Paris, France

**Keywords:** fecal microbiota transplantation, *Dientamoeba fragilis*, *Clostridioides difficile*, patient safety, treatment efficacy, shotgun sequencing, donor screening

## Abstract

*Dientamoeba fragilis* is a protozoan of the human digestive tract, yet its transmission and pathogenic role remain poorly understood. This study aimed to evaluate its impact on the efficacy and safety of fecal microbiota transplantation (FMT) in treating recurrent *Clostridioides difficile* infection (rCDI). This longitudinal cohort study analyzed stool samples from FMT donors and recipients pre-treatment and at 2 and 8 weeks post-FMT. All samples were retrospectively tested using real-time PCR. Shotgun metagenomics was also performed on selected donor–recipient pairs to explore transmission. CDI cure rates, gastrointestinal adverse events (AEs), and serious adverse events (SAEs) were assessed prospectively. A total of 53 FMT were analyzed (179 samples), with 23 (43%) derived from *D. fragilis*-positive donor stool (4 of 10 donors, 40%). Four of 52 recipients (18.2%), initially negative and who received treatment from positive donors, tested positive post-FMT. Shotgun metagenomics could not definitely confirm transmission due to the lack of a good reference genome. No significant differences in efficacy, AE, or SAE were observed between FMT from *D. fragilis*-positive versus -negative donors, even in immunocompromised patients. No SAEs were attributed to FMT. *D. fragilis* may be transmitted via FMT without evidence of short-term clinical impact. Consequently, RT-PCR detection should be interpreted cautiously in the context of donor exclusion decisions.

## 1. Introduction

Since 2014, fecal microbiota transplantation (FMT) has been the recommended treatment for recurrent *Clostridioides difficile* infections (rCDI), with success rates up to 85% and improved patient survival [1,2,3,4,5]. FMT aims to therapeutically modulate the recipient’s gut microbiome.

For safety reasons, donors undergo rigorous selection procedures, including multiple clinical and biological screenings, to minimize the risk of transmitting pathogens and potential diseases associated with gut microbiota [6]. Following this selection process, only 3–10% of candidates qualify as eligible fecal microbiota donors, limiting the availability and accessibility of this proven beneficial treatment [7,8].

*Dientamoeba fragilis* is an intestinal protozoan belonging to the *Trichomonadidae* order, even though it lacks flagella [9]. *D. fragilis* has been detected worldwide with prevalence rates ranging from 0.2% to 82%, and often exceeding 20% in molecular epidemiological studies [10]. However, its route of transmission remains poorly understood. The pathogenicity of *D. fragilis* remains debated, with limited evidence linking it to gastrointestinal symptoms such as diarrhea and abdominal pain [11].

FMT serves as a valuable model for studying the impact of controversial fecal microorganisms, providing direct exposure to donor gut microbiota in recipients. For example, *Blastocystis hominis*, another protist with debated pathogenicity, was removed from the donor exclusion list following a study by Teerver et al., which demonstrated no adverse consequences in recipients from *B. hominis*-positive donors [12]. As a result of this study, the 5th edition of the Guide to the Quality and Safety of Organs for Transplantation no longer lists *B. hominis* as an exclusion criterion for donors, unlike *D. fragilis*. The transmission and impact of *D. fragilis* during FMT, however, still require further investigation.

To date, real-time PCR (RT-PCR) for *Dientamoeba fragilis* is not included in routine donor screening at Lausanne University Hospital, the only center performing FMT in Switzerland. Donor screening for protists relies exclusively on direct microscopic examination. The primary objective of our study was to assess the prevalence of *D. fragilis* in stool samples from FMT donors and their paired recipients, both before and after FMT, using RT-PCR. Additionally, we aimed to investigate whether the presence of *D. fragilis* affects the efficacy of FMT or is associated with adverse events post-FMT.

## 2. Materials and Methods

### 2.1. Study Design and Setting

This observational cohort study was conducted at Lausanne University Hospital (CHUV) in Switzerland. From January 2023 to December 2024, all eligible patients receiving FMT as part of routine clinical care for rCDI and their corresponding donors involved in the production of FMT batches used for treatment were prospectively included. Molecular screening for *D. fragilis* was retrospectively performed using RT-PCR-based detection methods on stool samples collected from donors and from their paired recipients before and after FMT. The patient sample size was determined based on pragmatic considerations, and inclusion followed a total enumerative sampling approach, encompassing all patients who met the eligibility criteria over a two-year period. Study data were collected from patients’ electronic health records and were documented and managed using REDCap (version 14.6.11) electronic data capture tools hosted at CHUV. This manuscript was prepared in accordance with the STROBE statement for cohort studies [13].

### 2.2. Study Participants

#### 2.2.1. Patients–rCDI

Eligibility criteria required patients to be at least 18 years old and to have undergone FMT, with the indication aligned with the European Society of Clinical Microbiology and Infectious Diseases (ESCMID) guidelines for Clostridioides difficile treatment [1].

Patients received at least 10 days of pretreatment with fidaxomicin or vancomycin. FMT was administered to inpatients either via endoscopy (colonoscopy or jejunostomy) following bowel lavage with 2 L of macrogol solution the day prior or through oral ingestion of 20 capsules per day over two consecutive days (40 in total). In severe cases, the dosage was increased to 30 capsules per day (60 in total).

Patient variables were standardized, including age (continuous variable), sex (female or male), and immunosuppression status (binarized). Severe immunosuppression status was defined as current or foreseeable neutropenia (<500 neutrophils/µL) within the next 14 days; scheduled or recent (<100 days) allogeneic stem cell transplantation; active graft-versus-host disease (GvHD) requiring immunosuppressive treatment; or ongoing chemotherapy.

To assess the clinical success of FMT, cure was defined as the absence of CDI recurrence at 8 weeks post-FMT. Criteria for severe, complicated, and recurrent CDI followed the ESCMID guidelines [1]. Gastrointestinal adverse events (AEs) following FMT, including bloating, nausea, abdominal pain, and changes in bowel movements, were prospectively monitored and documented at weeks 2 and 8. Follow-up assessments were conducted via phone at 6 months, 12 months, and annually for up to 5 years.

Serious adverse events (SAEs) were evaluated in accordance with the ICH E2A guidelines [14]. The causal relationship between FMT and SAE was evaluated using categories defined in the Fecal Microbiota Transplant National Registry of the American Gastroenterological Association (AGA) [15].

#### 2.2.2. Donors

All microbiota donors were healthy, unpaid volunteers aged 18 to 50 years and were submitted to a detailed biological screening (Appendix A). Stool donations were collected at the CHUV FMT center and processed individually, without pooling between donations or donors (see Appendix B for detailed FMT production). The donors included in this study were those who provided stool used in the production of the treatment for the enrolled patients. In all samples, direct microscopic examination for *D. fragilis* yielded negative results.

### 2.3. Native Stool Collection, D. fragilis Detection

The donor’s native stool used for treatment was collected prior to treatment production. Stool samples from FMT recipients were obtained 24–48 h before FMT, as well as at 2 and 8 weeks post-FMT, as part of routine care for traceability purposes. All samples were aliquoted and biobanked at −80 °C (BIOSTOOL, BB_041). The diagnosis of *D. fragilis* was conducted using two in-house RT-PCRs at the Diagnostic Center of Swiss Tropical and Public Health Institute (Swiss TPH) in Allschwil (see Appendix B for detailed detection methods). A cut-off cycle threshold (Ct) of <40 was used for both PCRs. RT-PCR assays were used as established diagnostic tools with internal, positive, and negative controls; the study was not designed to perform analytical validation or assess inter-assay variability. Direct microscopic examination of donor stool was systematically negative in all cases. The analysis of shotgun metagenomics sequences of positive paired donor and recipient stools was performed at the Genomics and Metagenomics laboratory of the CHUV (see Appendix B for detailed methods).

### 2.4. Outcomes

The primary outcome was the assessment of the prevalence of *D. fragilis* in stool samples using RT-PCR from both donors and corresponding recipients before FMT, and at weeks 2 and 8 post-FMT. Secondary outcomes included comparing the efficacy, gastrointestinal AEs, and SAEs associated with FMT, depending on the *D. fragilis* status of donor batches.

### 2.5. Statistics

For the primary objective, the prevalence of *D. fragilis*-positive RT-PCR in donors and recipients was expressed as rates. With regard to the secondary objectives, the analysis focused on identifying differences in CDI recurrence rates and gastrointestinal AE and SAE between recipients of *D. fragilis*-positive and *D. fragilis*-negative treatments, using chi-square or Fisher’s exact test. A *p*-value below 0.05 was considered indicative of statistical significance in all comparisons. All analyses were performed using RStudio, version 4.2.1. No imputation was performed for missing data. All analyses were conducted on available cases only.

## 3. Results

### 3.1. RT-PCR Detection of D. fragilis in Stools from Donors and FMT Recipients

A total of 179 stool samples were analyzed by RT-PCR for *D. fragilis*, including 37 samples from 10 donors and 142 samples from 47 recipients. All donors and recipients were of European ethnic origin. The characteristics of the study population are summarized in Table 1. Details of recipients’ characteristics, clinical follow-up, RT-PCR results, and FMT data are provided in Supplementary Table S1.

*D. fragilis* was detected in the stool of 4 out of 10 donors (40%), with Ct values for RT-PCR ranging from 19.3 to 36.1 (Table 2). Fecal material from the 10 donors was used to produce a total of 233 oral FMT preparations (9320 capsules) and 54 suspensions. Batches derived from *D. fragilis*-positive donor stool accounted for 46 of the 233 oral preparations (1840 capsules, 19.7%) and 37 of the 54 suspensions (68.5%). In total, 53 FMT treatments were administered, of which 23 (43%) originated from *D. fragilis*-positive donor stool.

Prior to FMT, *D. fragilis* was negative in 52 of 53 (98%) recipient stool samples. One recipient (1/47, 2%) tested positive before FMT and received a treatment coming from *D. fragilis*-positive donor stool. There was a significant difference in the prevalence of *D. fragilis* between donors and recipients in our sample (*p* < 0.05). Among the 22 patients who tested negative before FMT and received stool from *D. fragilis*-positive donors, only four (18.2%) tested positive by RT-PCR at day 15 post-FMT, all of whom remained positive at week 8. All four patients received FMT via colonoscopy, with transplants thawed using a standardized warm water bath method (34.5 °C). Despite varying immunosuppression statuses, these patients tolerated the procedure well and were considered cured at the 8-week follow-up. The *D. fragilis* PCR Ct values in stool samples from positive donors used for their treatments ranged from 19.3 to 31.6. In positive recipients’ stool, Ct ranged from 21 to 34.5. Notably, four other patients who received FMT from the same donor batches under identical conditions did not test positive for *D. fragilis* at any point during follow-up.

Shotgun metagenomic sequencing was performed on one stool sample from donor DL01 and two samples from the FMT recipient RL42, as this pair presented the highest *D. fragilis* concentration (lowest Ct). From the 36 to 51 million reads generated per sample, only 19 (DL01), 380 (RL42 D15), and 31 (RL42 W8) reads mapped to the available *D. fragilis* transcriptome. Due to the extremely low number of mapped reads, we were unable to confirm with confidence the transmission of *D. fragilis* from donor to recipient.

### 3.2. Efficacy at 8 Weeks According to D. fragilis Positivity of the Donors

At week 8, CDI resolution was achieved in 44/47 (93.6%) patients after a single FMT, increasing to 46/47 (97.9%) after two FMTs and 47/47 (100%) after three. Notably, all post-FMT CDI recurrences occurred in recipients of stool from *D. fragilis*-negative donors. The presence of *D. fragilis* in donor stools had no impact on FMT efficacy. Although all observed CDI recurrences occurred in recipients of *D. fragilis*-negative donor stool, this difference did not reach statistical significance (*p* = 0.12). (Table 1). The median follow-up at the time of analysis was 13.3 months (range 3–25). Even though the cohort included individuals with significant morbidity and severe immunosuppression (see Supplementary Table S1), no new episodes of CDI were reported during the studied period after the 8-week follow-up.

### 3.3. AE and SAE at 15 Days and 8 Weeks Post FMT

Patients could experience multiple gastrointestinal AEs associated with a single FMT. A total of 53 gastrointestinal AE and 12 SAE were recorded and are detailed in Supplementary Table S1. None of the SAEs were related to FMT. Four SAEs (33%) occurred in recipients with transplants from *D. fragilis*-positive donors. There were no significant differences in the incidence of gastrointestinal adverse events or severe adverse events between patients treated with FMT from *D. fragilis*-positive or -negative donors (Table 3).

## 4. Discussion

The framework of FMT represents an excellent opportunity to study the debated pathogenicity of the enteric protozoan *D. fragilis*. To evaluate the potential impact of *Dientamoeba fragilis* colonization on gastrointestinal health, we retrospectively applied RT-PCR to stool samples from donors and their paired FMT recipients that had originally been screened by direct microscopic examination, and assessed associated mid-term clinical symptoms. To our knowledge, this is the first report of *D. fragilis* detection by RT-PCR in stool samples of previously negative recipients, following FMT. Although many donors tested positive for *D. fragilis* (40%), the new detection of this protozoan in recipients post-FMT via RT-PCR was rare, occurring in only 4 out of 22 cases. As previously described, the marked difference in RT-PCR prevalence between donors and recipients may reflect the contrast between healthy individuals and patients with severe gut dysbiosis. Neither the safety, as measured by the occurrence of AEs, nor the efficacy of FMT was affected by the detection of *D. fragilis* donors within this cohort. Although all CDI recurrences occurred in recipients of *D. fragilis*-negative donor stool, this difference did not reach statistical significance and should be interpreted cautiously. Given the limited sample size, this observation is hypothesis-generating only and does not support a protective effect of *D. fragilis.*

The life cycle of *D. fragilis* remains incompletely understood. While rare putative cyst and precyst forms have been observed in clinical specimens, the precise modes of human transmission remain unclear [9,16]. While direct fecal–oral transmission has yet to be confirmed [9], our results are suggestive of some level of transmission by this route and may support the hypothesis of the existence of a cyst stage in *D. fragilis* [17]. This finding, however, is contrary to previous studies, which found no evidence of *D. fragilis* transmission from donor to recipient via FMT in rCDI patients [18]. Moreover, the lack of a reproducible animal model for *D. fragilis* infection has significantly hindered research into its biology and pathogenicity.

Given the fragility of this protozoan, its survival during FMT production is questionable. As demonstrated by Hurych et al., a dramatic decrease in the viability of this protist after deep freezing is measured, with no viable organisms detectable in culture media after a single freeze–thaw cycle [19]. It is important to emphasize that although RT-PCR is a highly sensitive screening tool, its detection of DNA does not provide information about the viability or infectivity of the organism. Throughout this manuscript, *D. fragilis* RT-PCR positivity is interpreted as molecular detection and does not equate to confirmed colonization. However, the persistent finding of DNA for several weeks following FMT may suggest the possibility of colonization. Our findings, showing that 81.8% of recipients remained RT-PCR negative after receiving a *D. fragilis*-positive FMT, along with existing evidence, suggest that *D. fragilis* may only occasionally remain viable in the context of FMT. Notably, all instances of potential transmission occurred in recipients who received FMT via colonoscopy, suggesting that the viability of *D. fragilis* may depend on the route of administration or the type of preparation used.

While we attempted a shotgun sequencing approach to confirm the donor-to-recipient transmission of *D. fragilis* via FMT, the low concentration of the protozoan in stools, as well as the lack of a good reference genome for *D. fragilis*, complicated the analysis. The number of *D. fragilis* sequences recovered was too low to assert the genetic similarity of donor and FMT recipient strains. While unlikely, the four recipients who tested positive may also have been colonized by another natural source within 15 days post-FMT.

However, the critical question is not merely whether transmission occurs, but what the clinical consequences for recipients may be. Indeed, the pathogenicity of *D. fragilis* remains controversial. While it has been associated with gastrointestinal symptoms such as diarrhea and abdominal pain, the evidence supporting this link is inconsistent. A randomized controlled trial in Denmark by Röser et al. evaluated metronidazole for *D. fragilis* infections in 96 children, finding no significant symptom improvement over placebo, though parasitological eradication was more frequent in the metronidazole group [20]. More recently, a case–control study found no significant association between the detection of *D. fragilis* and the development of gastrointestinal symptoms [21]. Similarly, several case–control studies have failed to establish a link between *D. fragilis* and conditions such as irritable bowel syndrome (IBS) or celiac disease, aligning with findings for *B. hominis* [22,23,24].

Advances in molecular detection have demonstrated that *D. fragilis* is commonly found in asymptomatic individuals, consistent with the results observed in our donor cohort. A Danish study found *D. fragilis* prevalence at 43% in adults and 63% in children, indicating its high occurrence in the general population [25]. Likewise, in our cohort, stool donors—selected based on stringent safety criteria—frequently tested positive for *D. fragilis*. These findings support the hypothesis that *D. fragilis* may be a commensal organism within the normal gut microbiota, rather than a pathogenic entity. In our cohort, the low prevalence of these microorganisms in patients with dysbiosis before FMT further supports this model (2% in recipients versus 40% in healthy donors, *p* < 0.05) and even suggests that *D. fragilis* colonization may rather reflect gastrointestinal health.

This study has several limitations. First, the pragmatic sample size limited statistical power, increasing the risk of type II error, particularly for secondary outcomes. Second, multiple FMTs were derived from the same donors, introducing non-independence of observations. Third, as already mentioned, RT-PCR detection does not provide information on organism viability or true colonization. Fourth, shotgun metagenomic sequencing was exploratory, limited to a single donor–recipient pair with the highest RT-PCR signal, and lacked biological replication. Finally, FMT represents an artificial exposure model involving various modifications to the original stool sample. Consequently, the absence of clinical symptoms following FMT should not be interpreted as definitive evidence of non-pathogenicity of *D. fragilis* under natural transmission conditions. These findings may not be generalizable beyond patients with recurrent CDI undergoing FMT in a highly controlled clinical setting.

Recruiting stool donors for FMT is inherently challenging due to stringent screening criteria. As noted, *D. fragilis* is commonly found in asymptomatic individuals and is often a reason for donor exclusion [8]. In our study, had we applied *D. fragilis* positivity as an exclusion criterion, 40% of potential donors would have been excluded, resulting in the discarding of 287 FMT treatments derived from these donors. Given the complexity of donor selection, this would have significantly compromised the feasibility of producing this treatment and would have deprived patients of valuable treatments, as all recipients of *D. fragilis*-positive FMT in this study were cured. Our data suggest that *D. fragilis* colonization in donors, comparable to what has been demonstrated for *B. hominis* by Terveer et al., did not adversely affect recipient outcomes, including in high-risk patients [12]. This further supports the reconsideration of *D. fragilis* as an exclusion criterion for FMT donors.

## 5. Conclusions

Although *D. fragilis* has traditionally been considered pathogenic, its role in human health remains debated. FMT offered a valuable model for evaluating the pathogenicity of such protozoans with unclear or controversial clinical significance. Our findings suggest that FMT derived from *D. fragilis*-positive donor stools does not lead to gastrointestinal adverse events or severe outcomes in recipients, including those who are severely immunocompromised. These findings suggest that exclusion of donors based solely on RT-PCR detection of *D. fragilis* may warrant reconsideration, in line with the precedent set for *B. hominis* in the EDQM guidelines. Rationalizing donor screening processes could improve the availability of FMT without compromising safety, provided that appropriate clinical follow-up of recipients is ensured.

## Figures and Tables

**Table 1:** Demographic characteristics of fecal microbiota transplantation recipients and comparison between transfer of *D. fragilis*-positive and -negative donor stool.

Characteristics	*D. fragilis*-Negative Donor Stool ^a^	*D. fragilis*-Positive Donor Stool ^a^	*p*-Value ^b^
Age, years	73 (44, 79)	57 (52, 71)	0.2
Sex			>0.9
Male	73% (22/30)	74% (17/23)	
Female	27% (8/30)	26% (6/23)	
Severe CDI, yes	50% (15/30)	48% (11/23)	0.9
Immunosuppression, yes	27% (8/30)	43% (10/23)	0.2
Severe immunosuppression, yes	6.7% (2/30)	8.7% (2/23)	>0.9
Route of FMT administration			0.05
Capsules	70% (21/30)	43% (10/23)	
Colonoscopy	30% (9/30)	48% (11/23)	
Jejunostomy	0% (0/30)	8.7% (2/23)	
FMT efficacy			0.12
Cured	87% (26/30)	100% (23/23)	
Recurrence	13% (4/30)	0% (0/23)	

Abbreviations: CDI, *Clostridioides difficile* infection; FMT, fecal microbiota transplantation. ^a^ Median (Q1, Q3); % (n) ^b^ Wilcoxon rank sum test; Fisher’s exact test; Pearson’s *χ*^2^ test.

**Table 2:** Fecal microbiota transplantation with *D. fragilis*-positive donor stool and recipient *D. fragilis* status.

Donors			Recipients					
Donor ID	*D. fragilis* Ct Value	Storage Duration Pre-FMT ^a^, Days	Patient ID	*D. fragilis* Status Pre-FMT	*D. fragilis* Status D15 Post-FMT	Ct Value D15	*D. fragilis* Status W8 Post-FMT	Ct Value W8
DL01	23	237	RL01	Pos	Neg	n/a	Pos	30.6
DL01	23	222	RL02	Neg	Neg	n/a	Neg	n/a
DL01	23	217	RL03	Neg	Neg	n/a	Neg	n/a
DL01	23	168	RL05	Neg	Neg	n/a	Neg	n/a
DL01	23	204	RL05	Neg	Neg	n/a	Neg	n/a
DL01	23	162	RL07	Neg	Neg	n/a	Neg	n/a
DL01	23	203	RL08	Neg	Neg	n/a	Neg	n/a
DL01	23	35	RL10	Neg	Neg	n/a	Neg	n/a
DL01	27.2	239	RL35	Neg	Neg	n/a	Neg	n/a
DL01	23	257	RL36	Neg	Neg	n/a	Neg	n/a
DL01	23	384	RL38	Neg	Pos	25	Pos	21
DL01	19.3	370	RL42	Neg	Pos	21	Pos	21
DL01	19.3	377	RL45	Neg	Neg	n/a	Neg	n/a
DL01	23	729	RL47	Neg	Neg	n/a	Neg	n/a
DL02	23.13	202	RL04	Neg	Neg	n/a	Neg	n/a
DL02	23.13	246	RL37	Neg	Neg	n/a	Neg	n/a
DL02	23.13	379	RL46	Neg	Pos	27	Pos	26
DL03	32.5	260	RL06	Neg	Neg	n/a	Neg	n/a
DL03	31.6	106	RL27	Neg	Pos	33.6	Pos	34.5
DL03	31.6	462	RL43	Neg	Neg	n/a	Neg	n/a
DL03	31.6	497	RL44	Neg	Neg	n/a	Neg	n/a
DL05	36.1	714	RL11	Neg	Neg	n/a	Neg	n/a
DL05	30.5	702	RL11	Neg	Neg	n/a	Neg	n/a

Abbreviations: Ct, cycle threshold; FMT, fecal microbiota transplantation; ID, identification; n/a, not applicable; Neg, negative; Pos, positive; D15, 15 days post-FMT; W8, 8 weeks post-FMT. ^a^ Time elapsed between stool donation and FMT administration.

**Table 3:** Adverse events occurring at least once during follow-up and comparison between *D. fragilis*-positive and -negative donor stool recipients.

Gastrointestinal Adverse Events	*D. fragilis*-Negative Donor Stool ^a^	*D. fragilis*-Positive Donor Stool ^a^	*p*-Value ^b^
GI AE undif.	73.3% (22/30)	60.1% (14/23)	0.3
Constipation	7% (2/30)	8.7% (2/23)	>0.9
Diarrhea	16.7% (5/30)	4.3% (1/23)	0.5
Nausea	7% (2/30)	21.7% (5/23)	0.2
Abdominal pain	33.3% (10/30)	39.1% (9/23)	0.6
Bloating	10% (3/30)	21.7% (5/23)	0.3
Abdominal disconfort	3.3% (1/30)	8.7% (2/23)	0.6
Altered bowel habits	26.7% (8/30)	13% (3/23)	0.12
Other	10% (3/30)	4.3% (1/23)	>0.9
SAE	20% (6/30)	17.4% (4/23)	0.7

Abbreviations: GI, gastrointestinal; AE, adverse events; undif., undifferentiated; SAE, serious adverse events. ^a^ % (n). ^b^ Fisher’s exact test.

## Data Availability

The data presented in this study are openly available in [Mendeley data, https://data.mendeley.com/datasets/3f37czt42n/1] at [https://doi.org/10.17632/3f37czt42n.1].

## Supplementary Materials

The following supporting information can be downloaded at: https://www.mdpi.com/article/10.3390/microorganisms14010217/s1, Table S1: Source data.

## Abbreviations

The following abbreviations are used in this manuscript:

AEs	Adverse Events
AGA	American Gastroenterology Association
*B. hominis*	*Blastocystis hominis*
CDI	*Clostridioides difficile* infection
CHUV	Centre Hospitalier Universitaire Vaudois
Ct	Cycle Threshold
*D. fragilis*	*Dientamoeba fragilis*
EDQM	European Directorate for the Quality of Medicines & HealthCare
ESCMID	European Society of Clinical Microbiology and Infectious Diseases
FMT	Fecal Microbiota Transplantation
GvHD	Graft-versus-Host Disease
IBS	Irritable Bowel Syndrome
ICH E2A	International Conference on Harmonisation, article E2A (Clinical Safety)
ID	Identifier
rCDI	Recurrent CDI
REDCap	Research Electronic Data Capture software
RT-PCR	Real-Time Polymerase Chain Reaction
SAEs	Serious Adverse Events

## Appendix B

### Appendix B.1. FMT Production

The stool donation campaign at the CHUV Center lasts four weeks, commencing and concluding with a thorough screening process for donors according to ongoing Swissmedic regulations based on the current version of the European Directorate for the Quality of Medicines & HealthCare (EDQM).

Between 50 and 70 g of feces was used for FMT production of capsules and suspensions, respectively. For suspension preparation, the freshly collected donation was first diluted with 0.9% NaCl, then filtered using sterile non-woven gauze, and cryopreserved with glycerol to a final concentration of 10%. For capsule preparation, the freshly collected donation was diluted with 0.9% NaCl, filtered, and then centrifuged. After discarding the supernatant, the pellets were resuspended in glycerol, filtered, and double-encapsulated in modified-release and ready-to-administer 0 and 00 capsules (Capsugel^®^ DRCaps, Lonza, Stein, Switzerland).

FMT products were stored at −80 °C for up to 24 months. Before use, FMT suspensions were thawed either overnight at 2–8 °C or in a water bath at a maximum temperature of 35 °C for up to 90 min and then filtered and repackaged in syringes.

### Appendix B.2. Diagnosis of D. fragilis-RT-PCR

RT-PCR assays were performed using either native stool samples or stool samples preserved in BHIG. Preliminary tests on 10 reference samples with known presence of the pathogen demonstrated reproducible RT-PCR results regardless of the sample type.

DNA extraction: DNA was extracted using the DNA Mini kit protocol (Qiagen, 51306, Hilden, Germany) with minor modifications. For native stool samples, 100 mg of fresh stool was re-suspended in 1 mL of PBS. For feces preserved in BHIG medium, 500 uL of BHIG stool mixture was mixed with 1ml of phosphate-buffered saline (PBS), vortexed, and centrifuged (13,000× *g* for 10min) before the pellet was re-suspended in 500 µL PBS. Afterwards, both were centrifuged at 14,000 rpm for 5 min. The supernatant was removed, and the pellet re-suspended in 400 µL of ATL buffer and 40 µL of proteinase K, followed by an incubation at 56 °C for 2 h, interrupted by short mixing every 30 min. Later on, the samples were vortexed, and 200 µL of supernatant was further processed according to the kit protocol. DNA was eluted in 200 µL AE buffer and used for qPCR.

qPCR amplification: The 18S rRNA *Dientamoeba fragilis* specific RT-PCR protocol was conducted using TaqMan GenExpression Master Mix (Thermo Fisher, Basel, Switzerland, 4369016). The following sense primers, antisense primers and probe were used to amplify a 109 bp fragment of *D. fragilis*: (5′-to-3′ forward primer CAA CGG ATG TCT TGG CTC TT. 5′-to-3′ reverse primer AAT ACG CAA TGT GCA TTC AAA G [from Diagnostic center, Swiss TPH, self-designed], probe (hexachlorofluoroscein [HEX]-CAA TTC TAG CCG CTT AT-MGB-NFQ) (1) (Eurofins Genomics, Ebersberg, Germany).

The thermo profile for both qPCR on the QuantStudio 5 real-time PCR machine (Thermo Fischer) was 2 min at 50 °C and 10 min at 95 °C, followed by 40 cycles of 15 s at 95 °C and 1 min at 58 °C. On each plate of negative, internal, and positive controls (plasmids with a concentration of 103 containing an insert with the sequence of *D. fragilis*), real-time PCR products were included. A cut-off cycle threshold (Ct) of <40 was used for both PCRs.

### Appendix B.3. Metagenomics

For shotgun metagenomics sequencing, libraries were prepared using the Illumina DNA Prep kit before sequencing on a NextSeq 1000 (Illumina, San Diego, CA, USA), allowing us to retrieve between 35.8 and 50.97 million reads per sample. Given that no genome sequence is available for *D. fragilis*, the 6595 transcripts described by Barratt et al. [26] were used for read mapping using bowtie v2.5.4 with default parameters [27].
